# When the Mind Plays Tricks: LGI1 Encephalitis Mimicking Psychiatric Illness

**DOI:** 10.7759/cureus.86832

**Published:** 2025-06-26

**Authors:** Akshayaa K Aggarawal, Jithun v Varghese, Mansoor Gazi

**Affiliations:** 1 Internal Medicine, Walsall Manor Hospital, Walsall, GBR

**Keywords:** faciobrachial dystonic seizures (fbds), focal seizures, lgi1 antibody-mediated autoimmune encephalitis, normal eeg, psychiatric symptoms

## Abstract

Autoimmune encephalitis (AE) is a rare brain inflammation caused by the immune system. Leucine-rich glioma inactivated 1 (LGI1) antibody-associated AE is more common in older men and presents with memory loss, confusion, and brief seizures called faciobrachial dystonic seizures (FBDS). We report a 66-year-old man with hypertension and high cholesterol who presented with sleep-related spasms, confusion, and cognitive decline. Early magnetic resonance imaging (MRI) of the brain and electroencephalogram (EEG) were normal, delaying diagnosis. LGI1 antibodies were later detected in his blood. He received corticosteroid treatment, showing cognitive improvement and a decrease in the number of confusion episodes. The patient's family stated that the spasms were improved after treatment. This case highlights the need for early testing and treatment, even when initial findings are normal.

## Introduction

Autoimmune encephalitis (AE) is a diverse and increasingly recognized group of neuroinflammatory conditions caused by autoantibodies that target neuronal surfaces or connections (synapses) in the brain [1,2]. These conditions often exhibit a combination of brain-related and mental health-related symptoms, making early diagnosis challenging, particularly when brain scans and electroencephalogram (EEG) results appear normal.

Leucine-rich glioma-inactivated 1 (LGI1) antibody encephalitis is among the more common subtypes of AE, primarily affecting older male patients [3]. LGI1 is crucial for communication between nerve cells, as it interacts with voltage-gated potassium channels and other related proteins. When these pathways are disrupted, issues arise in brain areas involved in memory and emotions, especially the hippocampus and amygdala [4,5].

Patients typically present with subacute memory loss, personality changes, psychiatric disturbances, and seizures. Serum LGI1 antibodies are more reliable than cerebrospinal fluid (CSF) for diagnosis. Radiological investigations, such as computed tomography (CT) of the head and magnetic resonance imaging (MRI) of the brain, along with EEG, are often unremarkable [3]. Immunotherapy, including corticosteroids, intravenous immunoglobulin (IVIG), or plasmapheresis, is the mainstay of treatment, with additional immunosuppression considered in refractory cases. Early recognition and treatment are crucial for improving outcomes [5].

This case report highlights a diagnostically complex presentation of LGI1 encephalitis, emphasizing the need for clinical suspicion and repeat antibody testing in evolving neuropsychiatric illness.

## Case presentation

A 66-year-old forklift driver presented to Walsall Manor Hospital, Walsall, in March 2024 with a 10-day history of brief, non-convulsive episodes occurring once or twice daily, each lasting approximately 10 seconds. He reported experiencing spasms and episodic seizures one or two times a day over the past 10 days. These episodes were not tonic-clonic in nature and typically lasted around 10 seconds, making them likely myoclonic jerks. No pre-ictal changes or identifiable triggers were reported. During the episodes (ictal phase), there were no signs of tongue-biting, incontinence, altered consciousness, or overt confusion. Post-seizure, there was no post-ictal confusion. After these episodes, the patient experienced some dizziness and nausea.

His family noted progressive confusion and increasing forgetfulness over the past few months; however, there had been no change in his personality. Additionally, since February 2024, he described experiencing a peculiar “butterfly” sensation rising from his abdomen to his legs, often accompanied by a jolt-like electrical sensation traveling up through his body.

Despite the increasing frequency of these events and their impact on daily activities, multiple investigations yielded unremarkable results. He also showed signs of progressive confusion and memory loss over several months. His medical history included hypertension and hyperlipidemia, managed with simvastatin and ramipril, with no recent medication changes. He denied tobacco use but reported moderate alcohol intake on weekends.

Routine blood tests revealed that white blood cell count and neutrophils were slightly elevated, which may suggest a mild inflammatory or infectious process. The rest of the full blood count was normal, performed multiple times during his hospital stay and follow-up (Table 1).

**Table 1:** Full blood count. Hb: hemoglobin; WBCs: white blood cells; PLTs: platelets

Test	Result	Reference Range	Flag
Hb	133	130 - 180 g/L	Normal
WBCs	12.00	4.0 - 11.0 × 10⁹/L	High
PLTs	225	150 - 450 × 10⁹/L	Normal
Neutrophils	7.62	2 - 7.5 × 10⁹/L	High
Lymphocytes	3.49	1.5 - 4.5 × 10⁹/L	Normal
Monocytes	0.74	0.2 - 0.8 × 10⁹/L	Normal
Eosinophils	0.06	0.0 - 0.4 × 10⁹/L	Normal
Basophils	0.08	0.0 - 0.1 × 10⁹/L	Normal

Serum investigations revealed elevated alanine aminotransferase (ALT), a significant increase in gamma-glutamyl transferase (GGT), and mildly reduced total calcium levels. The mild GGT elevation may be attributed to regular, small amounts of alcohol consumed on weekends. The low calcium level could be due to vitamin D deficiency, although vitamin D was not tested. Other basic blood tests, including kidney function, as indicated by a normal glomerular filtration rate and serum electrolytes, were essentially within normal limits (Table 2).

**Table 2:** Biochemical investigations. EPI: Epidemiology Collaboration; eGFR: estimated glomerular filtration rate; ALP: alkaline phosphatase; ALT: alanine aminotransferase; GGT: gamma-glutamyl transferase

Test	Result	Reference Range	Flag
Sodium	135	133 - 146 mmol/L	Normal
Potassium	3.8	3.5 - 5.3 mmol/L	Normal
Urea	7.0	2.5 - 7.8 mmol/L	Normal
Creatinine	74	64 - 111 µmol/L	Normal
EPI eGFR	>90	>59 mL/min/1.73 m²	Normal
Total protein	64	60 - 80 g/L	Normal
Albumin	36	30 - 45 g/L	Normal
Globulin	28	15 - 50 g/L	Normal
Total bilirubin	11	5 - 26 µmol/L	Normal
ALP	66	30 - 130 IU/L	Normal
ALT	68	0 - 55 IU/L	High
GGT	196	12 - 64 U/L	High
Total calcium	2.16	2.20 - 2.60 mmol/L	Low
Adjusted calcium	2.25	2.20 - 2.60 mmol/L	Normal
Phosphate	0.93	0.8 - 1.5 mmol/L	Normal
Magnesium	0.86	0.7 - 1.0 mmol/L	Normal

At this point, after the initial workup was done and with no positive reports, the patient was initially thought to be experiencing psychiatric symptoms and was referred to Barberry Hospital, Birmingham, for evaluation by mental health teams for non-epileptic attacks. However, the symptoms did not subside.

Nevertheless, his clinical condition progressively deteriorated, marked by increasing impulsivity, aggression, and disinhibited behavior. Cognitive assessment revealed an Addenbrooke’s Cognitive Examination (ACE) score of 85/100, followed by a Mini-Mental State Examination (MMSE) score of 28/30, raising concern for a neurological deficit.

An AE antibody screen test from serum conducted in September 2024 detected positive LGI1 antibodies, confirming the diagnosis of LGI1 antibody-associated AE. Tests for antibodies against AMPA1, AMPA2, NMDA, and GABAB1/2 receptors were all negative, effectively excluding other common AE subtypes. Paraneoplastic antibodies were also not detected, making a paraneoplastic cause unlikely (Table 3).

**Table 3:** Antibody test (September 2024). IIF: indirect immunofluorescence

Antibody Test	Result
LGI1 antibody	Positive
AMPA1 receptor antibody	Negative
AMPA2 receptor antibody	Negative
NMDA receptor antibody	Negative
GABAB1/2 receptor antibody	Negative
Paraneoplastic antibody (IIF)	Negative

The patient's initial blood tests were positive, though a false positive result or lab error was considered a possibility. Therefore, the tests were repeated. Follow-up blood tests in October 2024 confirmed the presence of LGI1 antibodies, while all other AE antibodies remained negative (Table 4).

**Table 4:** Antibody test (October 2024)

Antibody Test	Result
LGI1 antibody	Positive
AMPA1 receptor antibody	Negative
AMPA2 receptor antibody	Negative
NMDA receptor antibody	Negative
GABAB1/2 receptor antibody	Negative

The autoimmune antibody tests showed a weakly positive antinuclear antibody (ANA) screen, which may indicate a mild or nonspecific autoimmune response but is not definitive for any autoimmune disease on its own. Both myeloperoxidase (MPO) antineutrophil cytoplasmic antibody (ANCA) and proteinase 3 (PR3) ANCA levels were within normal reference ranges, suggesting no active vasculitis or ANCA-associated autoimmune disorder, ruling out a vasculitic cause for this presentation (Table 5).

**Table 5:** Serum autoimmune profile. ANCA: antineutrophil cytoplasmic antibody

Test	Result	Reference Range
Antinuclear antibody screen	Weak positive	-
Myeloperoxidase (MPO) ANCA	<0.2	0 - 3.5 IU/mL
Proteinase 3 (PR3) ANCA	1.50	0 - 2 IU/mL

The CSF analysis showed clear, colorless fluid with very low white and red blood cell counts, indicating no significant inflammation or bleeding. The small number of red blood cells could have been due to trauma. Normal glucose and protein levels suggested no infection. No organisms were seen, and cultures showed no growth, ruling out bacterial infection. In summary, infections were ruled out as a cause of this presentation (Table 6).

**Table 6:** CSF report (November 2024). CSF: cerebrospinal fluid; WBCs: white blood cells; RBCs: red blood cells

CSF Findings	Result
Appearance	Clear, colourless fluid
WBCs	3 × 10⁶/L
RBCs	6 × 10⁶/L
Organisms	No organisms seen
Culture	No growth
CSF glucose	3.9 mmol/L

The CSF autoimmune antibody panel was entirely negative, including tests for encephalitis-related antibodies such as LGI1, NMDA, AMPA1/2, and others, making active AE in the CSF less likely (Table 7).

**Table 7:** CSF antibody test (November 2024). CSF: cerebrospinal fluid

CSF Antibody Test	Result
CSF encephalitis screen	Negative
CSF AMPA1/2 receptor antibody	Negative
CSF anti-DPPX antibody	Negative
CSF LGI1 antibody	Negative
CSF NMDA receptor antibody	Negative
CSF CASPR2 antibody	Negative
CSF GABAB1/2 receptor antibody	Negative
Paraneoplastic CSF antibody	Negative

Comprehensive infectious screening using polymerase chain reaction (PCR) and BioFire (BioFire Diagnostics, USA) detected no DNA or RNA from common pathogens known to cause meningitis or encephalitis, including herpes viruses, enteroviruses, and various bacteria. This effectively ruled out infectious causes for the patient’s symptoms (Table 8). 

**Table 8:** CSF viral screen test (November 2024). CSF: cerebrospinal fluid; PCR: polymerase chain reaction; RT-PCR: reverse transcriptase-polymerase chain reaction

CSF Test	Result
*Escherichia coli* K1 DNA (BioFire)	Not detected
*Haemophilus influenzae* DNA (BioFire)	Not detected
*Listeria monocytogenes* DNA (BioFire)	Not detected
*Neisseria meningitidis* DNA (BioFire)	Not detected
*Streptococcus agalactiae* DNA (BioFire)	Not detected
*Streptococcus pneumoniae* DNA (BioFire)	Not detected
Cytomegalovirus (CMV) DNA (BioFire)	Not detected
Enterovirus RNA (BioFire)	Not detected
Herpes simplex virus 1 DNA (BioFire)	Not detected
Herpes simplex virus 2 DNA (BioFire)	Not detected
Human herpesvirus 6 DNA (BioFire)	Not detected
Human parechovirus RNA (BioFire)	Not detected
Varicella zoster virus DNA (BioFire)	Not detected
*Cryptococcus neoformans/gattii *(BioFire)	Not detected
Herpes simplex type 1 DNA (PCR)	Not detected
Herpes simplex type 2 DNA (PCR)	Not detected
Varicella zoster DNA (PCR)	Not detected
Enterovirus RNA (RT-PCR)	Not detected
Parechovirus RNA (RT-PCR)	Not detected

**Figure 1:**
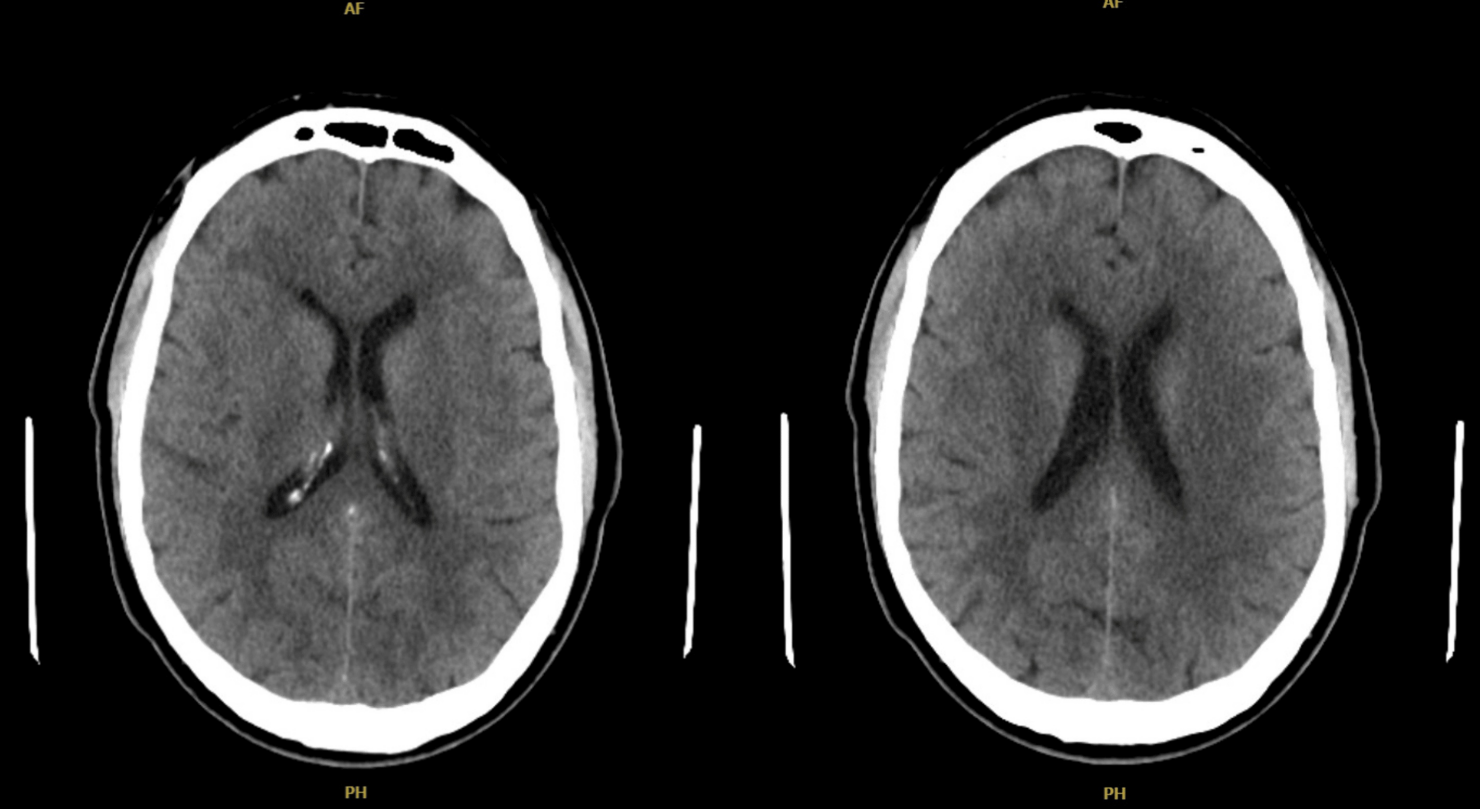
Axial CT of the brain showing normal brain parenchyma and ventricles. CT: computed tomography

Neuroimaging (CT head) was performed in September 2024, and it was normal. No focal intracerebral lesions, intracranial hemorrhage, or collections were seen (Figure 1). 

**Figure 2:**
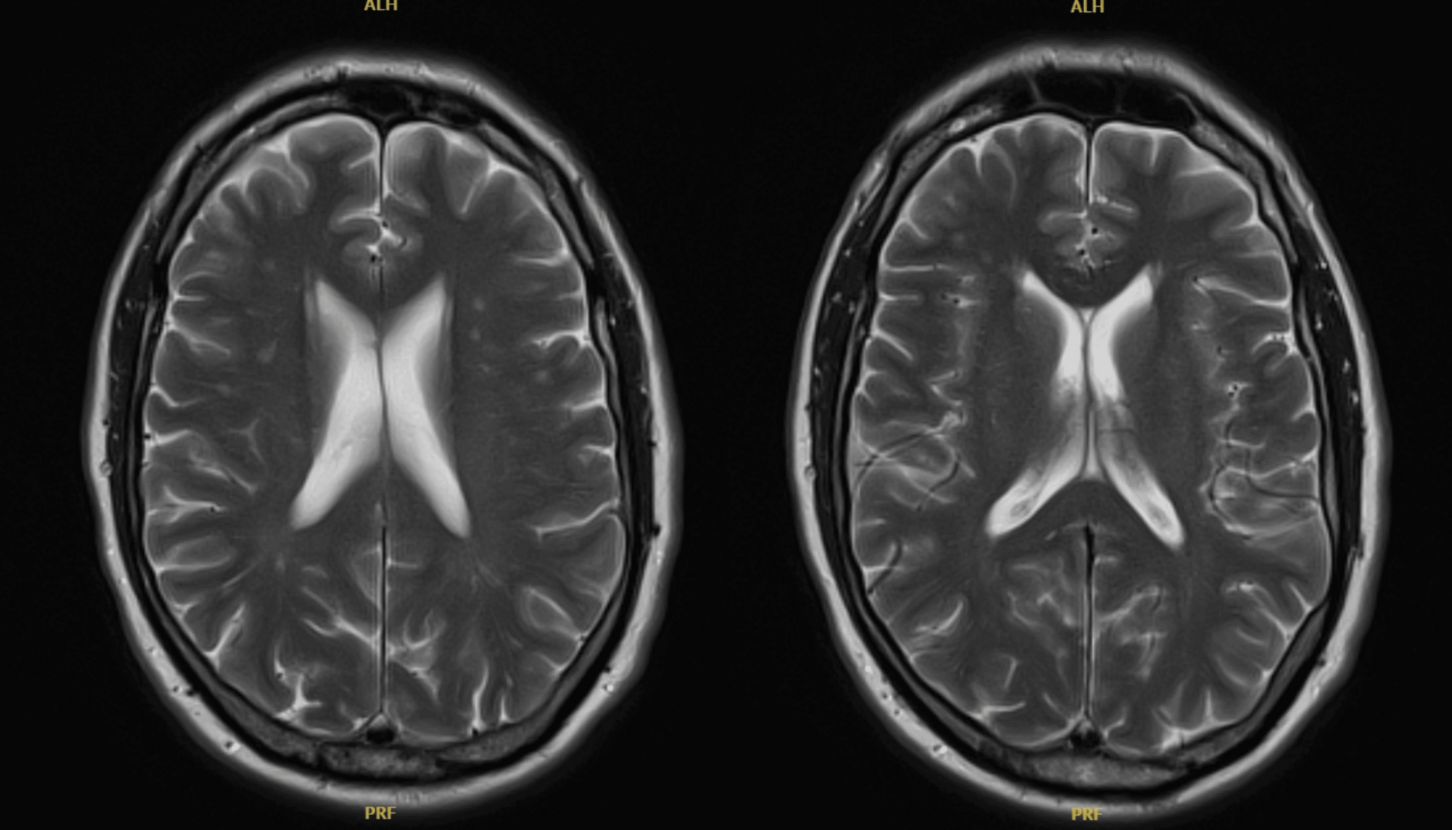
Axial brain MRI showing normal brain tissue and well-preserved ventricular structures. MRI: magnetic resonance imaging

**Figure 3:**
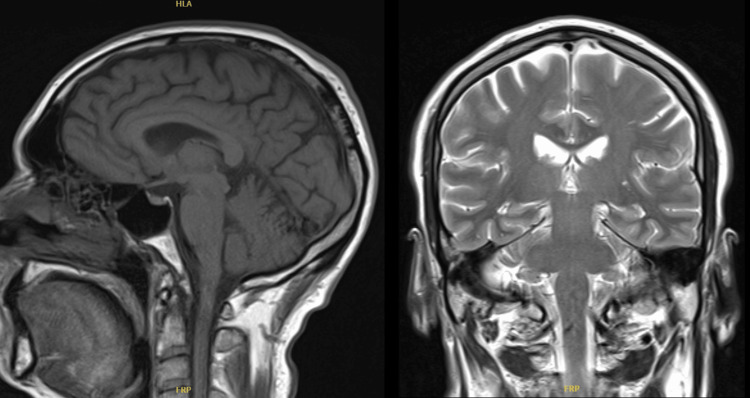
Brain MRI showing normal anatomy on both sagittal T1 (left) and coronal T2 (right) views. The ventricles are of normal size and shape, with no evidence of mass, hemorrhage, or structural abnormalities. The brain parenchyma, brainstem, and cerebellum appear intact and symmetrical, with no signs of oedema or lesions. Overall, the imaging is within normal limits. MRI: magnetic resonance imaging

The MRI of the brain conducted in May 2024 during this period revealed no focal intracranial pathology (Figures 2-3). 

**Figure 4:**
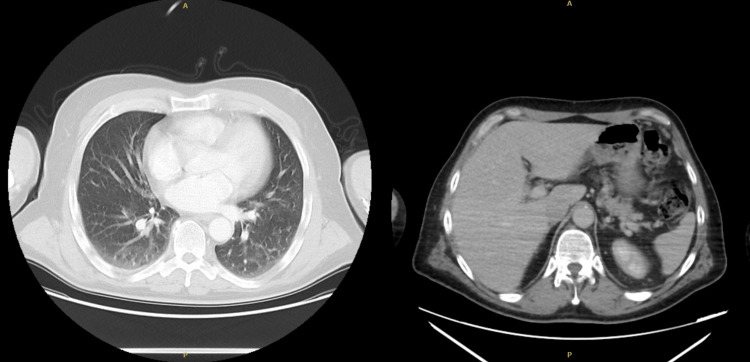
CT of the chest (left) and abdomen (right) showing normal lung parenchyma and no organomegaly. No signs of malignancy or abnormal enlargement are seen. CT: computed tomography

**Figure 5:**
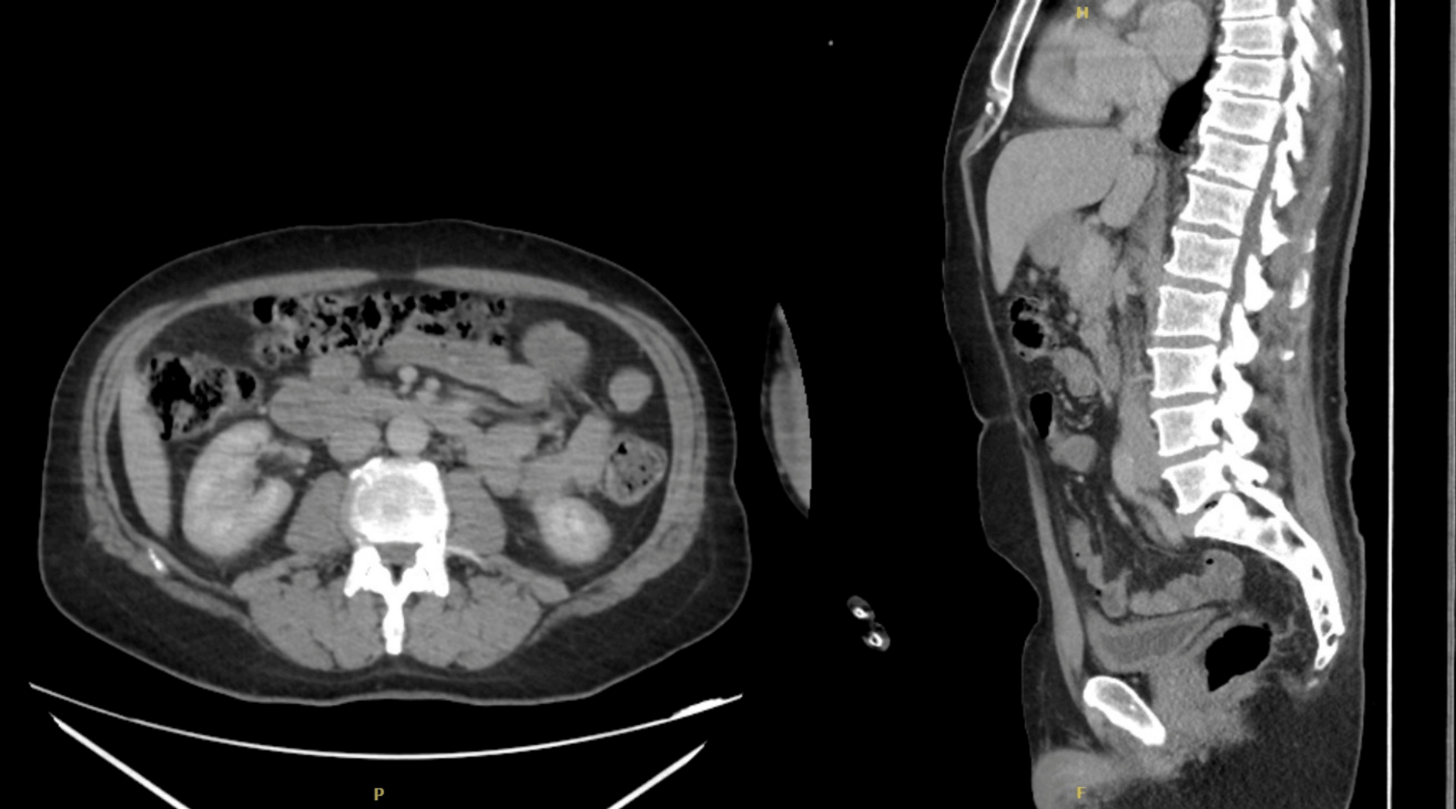
CT of the abdomen in axial (left) and sagittal (right) views showing normal findings. The kidneys, liver, spleen, and pancreas appear unremarkable. Bowel loops are normal, with no signs of obstruction. The spine is well aligned, and no masses or abnormal fluid collections are seen. No signs of malignancy are present. CT: computed tomography

A CT of the chest, abdomen, and pelvis identified mild prostatomegaly and showed no malignancy, concluding that there was no underlying malignancy, a common issue in this age group, as a potential cause of the symptoms (Figures 4-5).

The EEG showed no clear seizure activity. One of the typical attacks of the patient was recorded, and it showed no seizure activity on EEG, making epilepsy less likely.

Initial medical management included lamotrigine, titrated to target both seizure-like activity and behavioral symptoms. Following further clinical deterioration, the patient was admitted in December 2024, and further workup was done. At this time, the patient was diagnosed with AE and started on steroids. The initial treatment included intravenous methylprednisolone therapy, which on discharge was converted to oral therapy on a tapering dose. Subjective improvement in cognition was noted by his family, although prominent hallucinations and sleep disturbances persisted. Melatonin was prescribed to address the latter.

A cardiology evaluation ruled out a syncopal origin for the patient’s drop attacks, reinforcing the likelihood of a neurological basis.

As of February 2025, the patient continued to experience residual neuropsychiatric symptoms, including agitation, insomnia, and visual hallucinations. Despite these challenges, his cognitive performance remained relatively preserved. Overall, the patient showed clinical improvement and was regularly followed up by the neurology department (Table 9).

**Table 9:** Timeline of our case. EEG: electroencephalogram; MRI: magnetic resonance imaging

Month	Clinical Event
March 2024	Hospital admission for seizure-like episodes - treated as psychiatric episodes
April 2024	MRI and EEG done - normal
September 2024	Behavioural changes persisted
October 2024	LGI1 antibody was found positive
November 2024	LGI1 antibody positive - confirmed again
December 2024	Diagnosis confirmed, treatment initiated
February 2025	Improvement in clinical symptoms

## Discussion

This case illustrates the diagnostic complexity and clinical variability associated with LGI1 antibody-mediated AE, a relatively common but frequently underdiagnosed subtype of AE, particularly in older adults. Despite its increasing recognition, the diagnosis remains challenging due to its protean manifestations and often unremarkable early investigations [6]. In this patient, the diagnostic journey was prolonged, with several months elapsing from initial presentation to definitive diagnosis. A key challenge was the absence of classic seizure features, such as tongue biting, postictal confusion, or incontinence. The patient’s seizure-like episodes, characterized by a “butterfly” sensation, mild jerks, and occasional falls, were atypical and subtle, mimicking non-epileptic events or psychiatric disturbances. Moreover, normal findings on MRI and EEG, both early and repeated, further obscured the clinical picture. This is not unusual, as studies indicate that up to 50% of LGI1 AE cases may have normal MRI scans at the time of presentation, and EEGs frequently lack specific epileptiform activity, particularly in patients without faciobrachial dystonic seizures (FBDS). Similar atypical presentations have been reported, such as ictal piloerection and insomnia without cognitive symptoms, which further complicate the clinical picture [7]. Although FBDS are considered pathognomonic, they are not universally present. Literature shows that FBDS precede cognitive decline in only around 50% of cases [8].

The serological identification of LGI1 antibodies was pivotal in establishing the diagnosis. In contrast to other autoimmune encephalitides like anti-NMDA receptor encephalitis, where CSF antibody positivity is more common, LGI1 antibodies are typically more reliably detected in serum than in CSF, which was consistent with this case. Despite negative CSF studies, the positive serum LGI1 antibodies provided a definitive diagnostic clue and aligned with the patient’s limbic-predominant neuropsychiatric syndrome. In our case, diagnostic clarity was achieved only after serum LGI1 antibody testing, with CSF studies remaining negative, a common finding in LGI1 AE, as the antibodies are more reliably detected in the serum [9]. This underscores the importance of repeated autoimmune testing in patients with evolving symptoms despite normal radiological and electrophysiological findings [10].

The presence of neuropsychiatric symptoms such as disinhibition, hallucinations, and impulsivity mirrors other reported cases where AE was initially mistaken for psychiatric illness or early-onset dementia [6,11]. Early immunotherapy, especially corticosteroids, has been shown to improve outcomes and may prevent irreversible cognitive damage if initiated promptly [9,11].

The patient's response to oral prednisolone and adjunctive melatonin for sleep disturbances supports the existing body of evidence indicating that LGI1 AE is highly responsive to immunotherapy, although some patients may require escalation to agents such as rituximab or azathioprine in refractory cases [12]. 

## Conclusions

In summary, this case highlights the critical importance of maintaining a high level of clinical suspicion for AE, particularly LGI1 antibody-associated encephalitis, in older adults who present with subacute neuropsychiatric symptoms and atypical seizure activity. Diagnosis can be challenging, especially when imaging and EEG results are unremarkable, making early serum antibody testing essential. Clinicians should consider encephalitis in patients exhibiting subacute changes in behavior, memory, or cognition; new-onset psychiatric symptoms; unusual or focal seizures (such as FBDS seen in LGI1 encephalitis); and unexplained confusion or altered consciousness. Early recognition facilitates the timely initiation of immunotherapy, which can significantly improve outcomes, reduce the risk of lasting cognitive impairment, and prevent irreversible neurological damage. This case further underscores the value of a multidisciplinary care approach and continuous monitoring to optimize recovery and ensure long-term neurological health. 

## References

[1] Gole S, Anand A (2023). Autoimmune encephalitis. StatPearls [Internet].

[2] Lai M, Hughes EG, Peng X (2009). AMPA receptor antibodies in limbic encephalitis alter synaptic receptor location. Ann Neurol.

[3] van Sonderen A, Schreurs MW, de Bruijn MA (2016). The relevance of VGKC positivity in the absence of LGI1 and Caspr2 antibodies. Neurology.

[4] Bien CG, Vincent A, Barnett MH (2012). Immunopathology of autoantibody-associated encephalitides: clues for pathogenesis. Brain.

[5] Graus F, Titulaer MJ, Balu R (2016). A clinical approach to diagnosis of autoimmune encephalitis. Lancet Neurol.

[6] Wu H, Mei F, Liu L, Zhang L, Hao H, Zhang S (2021). Rare case of anti-LGI1 limbic encephalitis with rapidly progressive dementia, psychiatric symptoms, and frequently seizures: a case report. Medicine (Baltimore).

[7] Anandan S, Rajendran SS, Kumar JP, Shajee DS (2024). A stitch in time saves nine: a case of anti LGI1 encephalitis presenting as goose bumps. Neurol India.

[8] Sikka C​, Mahajan NS, Mahajan K, Mahajan R (2024). Anti-LGI1 autoimmune limbic encephalitis presenting with psychiatric manifestations - an under-recognized entity: a rare case report. Arch Biol Psychiatry.

[9] Kurukumbi M, Castillo JA, Shah T, Gupta R (2019). Rare case of anti-LGI1 limbic encephalitis with new onset epilepsy: a case report. Cureus.

[10] Agarwal V, Gongati N, Kachu R (2024). A case report on leucine rich glioma inactivated 1 antibody encephalitis. Int J Res Med Sci.

[11] Luster JD, Barasa A, Hoffman W (2022). Rapidly progressive dementia with recurrent seizures and hyponatremia: a case of LGI1 limbic encephalitis. Neuroimmunol Rep.

[12] Dalmau J, Graus F (2018). Antibody-mediated encephalitis. N Engl J Med.

